# The Mediating Role of Perceived Social Support in Family Cohesion and Disease Identity Among Chinese Adolescents and Young Adults with Type 1 Diabetes Mellitus: A Cross-Sectional Study

**DOI:** 10.3390/nursrep16020048

**Published:** 2026-01-30

**Authors:** Xiao Yang, Xiaofan Wang, Chunhui Zhang, Xian Zhang

**Affiliations:** School of Nursing and Health, Zhengzhou University, Zhengzhou 450001, China; zzu2024yx@163.com (X.Y.); wangxf202409@163.com (X.W.)

**Keywords:** adolescents and young adults, type 1 diabetes mellitus, family cohesion, perceived social support, disease identity

## Abstract

**Objective**: This study aimed to examine the association between family cohesion and disease identity in adolescents and young adults (AYAs) with type 1 diabetes mellitus (T1DM) and to test the mediating role of perceived social support in this relationship. **Methods**: From January 2025 to June 2025, a total of 222 AYA patients with T1DM were recruited from the Department of Endocrinology and Department of Pediatrics of four tertiary-level hospitals in Zhengzhou, Henan Province, China. The Disease Identity Questionnaire for AYA patients with T1DM, Family Cohesion Scale, and Perceived Social Support Scale were used. **Results**: Family cohesion was positively correlated with perceived social support and disease identity, and perceived social support was positively correlated with disease identity. Perceived social support played a partial mediating role in the association between family cohesion and disease identity (β = 0.391, *p* < 0.001), accounting for 63.6% of the total effect. **Conclusions**: Family cohesion is positively associated with perceived social support and disease identity in AYA patients with T1DM, with perceived social support playing a partial mediating role. This indicates that family cohesion shows both direct and indirect associations with patients’ disease identity. The study suggests that interventions aimed at enhancing family cohesion and perceived social support may inform strategies to improve patients’ disease identity, thereby potentially facilitating their psychosocial adaptation.

## 1. Introduction

Type 1 diabetes mellitus (T1DM) represents a growing global public health concern among AYAs (aged 10–24 years), with its incidence rising worldwide [1,2]. A recent study indicates that the incidence of T1DM in this population increased from 7.78 per 100,000 persons in 1990 to 11.07 per 100,000 in 2019 [3]. Although precise national data on the incidence of T1DM specifically for the 10–24-year-old age group in China are currently unavailable, existing evidence consistently points to a rapidly increasing epidemic trend. The overall annual incidence of T1DM across all age groups in China is 1.01 per 100,000 [4]. Regarding age distribution, the peak onset of T1DM in China is concentrated in AYAs [5]. A nationwide multicenter study involving 34 medical centers revealed that among newly diagnosed Chinese T1DM patients in adolescence and early adulthood, the incidence in the ≥10-year age group was significantly higher than that in the 5–10-year and <5-year groups, underscoring that AYAs constitute a high-risk phase [5].

AYAs is a critical period for identity formation. A diagnosis of T1DM compels individuals to integrate a “disease identity” into their self-concept [6]. Disease identity refers to the process by which patients incorporate the disease experience into their self-concept. This involves accepting the diagnosis, proactively disclosing their condition to significant others, remaining resilient against stigma, and consistently engaging in self-management [7,8]. As a psychological and behavioral characteristic, disease identity is closely associated with glycemic control outcomes [9]. A positive disease identity is crucial for patients’ treatment adherence, psychological well-being, and long-term prognosis [10].

However, studies indicate [11,12,13,14] that many AYA patients with T1DM experience identity conflict and a high prevalence of stigma, which can hinder the adaptive integration of diabetes into their personal identity. This often leads to neglected disease management and poor glycemic outcomes [15]. Consequently, facilitating the development of a positive disease identity has become a key objective in the management of AYA patients with T1DM.

The relationship between the family and the patient influences the patient’s perception of their disease identity [16]. Adolescents who receive consistent parental support achieve and sustain better adaptation in diabetes management [17]. In China, parents play a dominant role in the health management of AYA patients with T1DM [18]. A family environment characterized by high cohesion builds a solid foundation of psychological security for the patient through continuous emotional care and positive interactive feedback. Patients raised in such an environment are more likely to develop positive interpersonal cognitive patterns [19], which in turn establishes a psychological foundation for accepting their disease identity.

Perceived social support refers to the extent to which an individual subjectively feels supported by others or society [20]. When patients perceive a high level of social support, it signifies that their social environment holds an accepting and inclusive attitude toward their disease state. The study by Nunez-Baila et al. [21] found that AYA patients with T1DM, upon perceiving friendliness and support from their peers, were more willing to actively integrate into the group and had reduced concerns regarding their disease identity. Therefore, enhancing the level of patients’ perceived social support is of significant importance for the formation of a positive disease identity.

Although existing research has separately examined the association of family factors or social support with disease identity [22,23], the pathway through which family cohesion affects disease identity via internal psychological mechanisms remains unclear [16,21,24,25]. There is a particular lack of research investigating the mediating role of perceived social support in the relationship between family cohesion and disease identity among AYA patients with T1DM. This study employs the social support theory as its framework, primarily because it clearly elucidates the crucial role of perceived support in linking external environments with internal cognitive processes. The theory posits [26] that an individual’s perceived social support can promote physical and mental health through both main and buffering effects, which directly aligns with the variable relationships explored in this study. Consequently, based on this theoretical foundation, we hypothesize that family cohesion serves as a factor in generating high-quality perceived social support. Perceived social support, acting as an intermediary bridge in the buffering effect, translates family interactions into tangible support for the individual, which in turn further facilitates the development of a positive disease identity among AYA patients with T1DM.

**Figure 1:**
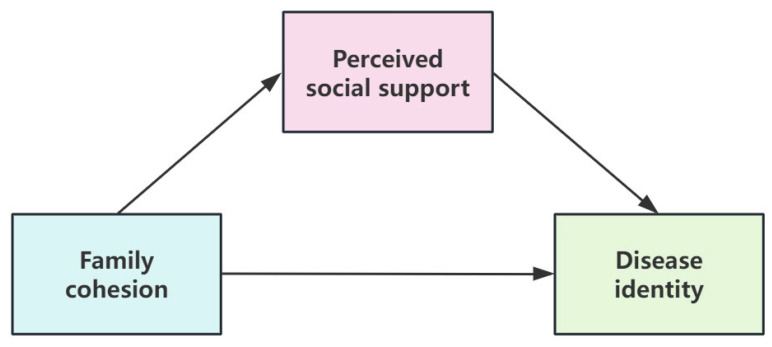
Research framework of the study.

Therefore, this study aimed to measure the associations among family cohesion, perceived social support, and disease identity, and to examine the mediating role of perceived social support in the association between family cohesion and disease identity among AYA patients with T1DM. Based on the research objectives and as illustrated in Figure 1, a mediating model was constructed to test the following hypotheses (H):

**Hypothesis** **1:** *Family cohesion is positively associated with disease identity among AYA patients with T1DM.*

**Hypothesis** **2:** *Perceived social is positively associated with disease identity among AYA patients with T1DM.*

**Hypothesis** **3:** *Family cohesion is positively associated with perceived social support among AYA patients with T1DM.*

**Hypothesis** **4:** *Perceived social support mediates the association between family cohesion and disease identity among AYA patients with T1DM.*

## 2. Methods

### 2.1. Design

This study used a quantitative, cross-sectional correlational design to explore the relationship between family cohesion, perceived social support, and disease identity among AYA patients with T1DM. This manuscript adheres to the Strengthening the Reporting of Observational Studies in Epidemiology (STROBE) statement for reporting observational research.

### 2.2. Participants/Sampling

Between January 2025 and June 2025, a convenience sample of AYA patients with T1DM was recruited from the Department of Endocrinology and Department of Pediatrics of four tertiary-level hospitals in Zhengzhou, Henan Province, China. Inclusion criteria: (1) Age between 10 and 24 years; (2) meeting diagnostic criteria for type 1 diabetes: diagnosed by an endocrinologist at a tertiary hospital in accordance with the Chinese guidelines for the Diagnosis and Treatment of Type 1 Diabetes [27]; (3) disease duration ≥3 months; (4) be conscious and able to communicate independently; (5) voluntary participation. Exclusion criteria: (1) Current acute complications such as ketoacidosis or hypoglycemia; (2) presence of psychological disorders or severe intellectual disability. The World Health Organization (WHO) defines Youth as individuals aged 15–24 years and Adolescents as those aged 10–19 years [28]. Given the partial overlap between these two age ranges, the broader cohort spanning 10–24 years is collectively termed adolescents and young adults (AYA). The selection of patients aged 10–24 years with type 1 diabetes as research subjects in this study is mainly based on two reasons. First, most patients in this age group in China are enrolled in schools and thus exhibit a certain degree of homogeneity. Second, considering the difficulty in sample size collection, this age delimitation helps obtain an adequate number of research samples within a feasible scope.

Based on the rule-of-thumb estimation method for sample size in cross-sectional studies, the sample size should be 5 to 10 times the number of variables [29]. This study included a total of 13 variables, encompassing a general information questionnaire, the Family Cohesion Scale, and the Perceived Social Support Scale. Accordingly, the sample size was calculated as 10 times the number of variables (13), resulting in 130. Considering an estimated 10% rate of invalid questionnaires, the minimum required sample size was adjusted to 145. Furthermore, as the planned subsequent analysis involves using the Bootstrap method for mediation analysis-a method for which a sample size of no less than 200 is typically recommended to ensure the stability of the results-this study ultimately distributed 230 questionnaires. A total of 222 valid questionnaires were collected, yielding an effective response rate of 96.5%.

### 2.3. Instruments

(1) General Data Collection

The general information questionnaire comprised two sections: Part One covered sociodemographic data including gender, age, education level, only child status, and family residence. Part Two addressed disease-related information such as duration of disease and glucose control methods.

(2) Family Cohesion Scale

The Family Cohesion subscale was adopted from the Family Cohesion and Adaptability Scale, originally developed by Olson et al. [30] and later translated and validated in Chinese by Zheng et al. [31]. This subscale specifically assesses the emotional bonds among family members, which aligns with the focus of the present study, rather than the family system’s adaptability. The subscale consists of 16 items rated on a 5-point Likert scale (1 = Not at all to 5 = Always). Higher total scores indicate greater family cohesion. In the Chinese validation study, the subscale demonstrated good reliability, with a Cronbach’s α of 0.85 and a test–retest reliability of 0.84 [31]. The Cronbach’s α in this study was 0.82.

(3) Perceived Social Support Scale (PSSS)

Jiang et al. [32] developed the PSSS by translating and revising the multidimensional social support scale created by Zimet et al. [33]. It serves as a tool to measure an individual’s perceived level of social support from multiple sources. This scale comprises three dimensions (family support; friend support; and other support) and 12 items. The scale employs a 7-point rating scale (1 = “completely disagree,” 7 = “completely agree”). Higher total scores indicate greater perceived social support from others. Scores are categorized as follows: 12–36 points = low perceived support level, 37–60 points = moderate perceived support level, 61–84 points = high perceived support level. The scale demonstrated a total Cronbach’s α coefficient of 0.93 [32]. It encompasses multidimensional social support as perceived by individuals and has been validated for reliable and valid application among adolescents [34]. The Cronbach’s α in this study was 0.94.

(4) AYA patients with T1DM Disease Identity Questionnaire

This study employed self-developed AYA patients with the T1DM Disease Identity Questionnaire. The scale was constructed following a standard procedure: an initial version (4 dimensions, 41 items) was drafted based on literature review and semi-structured interviews and then refined through two rounds of Delphi consultation with 15 experts. The scale-level content validity index (S-CVI/Ave) was 0.95, and the item-level content validity indices (I-CVI) ranged from 0.88 to 1.00, resulting in a pilot version (4 dimensions, 25 items). After pilot testing with 30 patients, two items were deleted based on item analysis. Exploratory factor analysis of the remaining 23 items (KMO = 0.833; Bartlett’s test, *p* < 0.001) extracted four common factors, which accounted for 62.88% of the total variance, with factor loadings ranging from 0.492 to 0.864. Two further items were removed, yielding a final 21 items version. Confirmatory factor analysis supported the four-factor structure, with good model fit (χ^2^/df = 1.215, GFI = 0.918, CFI = 0.984, TLI = 0.982, RMSEA = 0.031).

The final questionnaire comprises 21 items across four dimensions: Disease Identity, Identity in Interaction, Identity Expression, and Identity Selection. It employs a 5-point Likert scale, with total scores ranging from 21 to 105; higher scores indicate stronger disease identity. The overall Cronbach’s α was 0.861, and dimension-level α coefficients ranged from 0.820 to 0.884. Test–retest reliability was 0.973. For descriptive purposes, total scores were categorized into three groups (Low: 21–57; Medium: 58–77; High: ≥78) based on K-means cluster analysis; however, all primary inferential analyses utilized the continuous total scores.

### 2.4. Survey Methodology

The survey was conducted by four researchers who received standardized training prior to the study. Training covered questionnaire distribution, completion procedures, and key considerations. Before completion, patients were informed of the survey’s purpose, significance, and confidentiality principles, with assurances of anonymity. After obtaining consent, patients completed the questionnaire independently. For those unable to complete the questionnaire independently due to visual impairment or literacy limitations, researchers read each question aloud and completed the form truthfully on their behalf. Questionnaires were completed and collected on-site. Researchers verified completion status and reminded patients to fill in any omitted items. Data entry was performed by two individuals to ensure accuracy.

### 2.5. Statistical Methods

Data analysis was performed using SPSS 27.0 software. The normal distribution was evaluated with skewness and kurtosis values (±2) [35]. Categorical variables were described using frequency and percentage, while continuous variables (including summated Likert-scale scores) [36] were described using mean ± SD. We employed *t*-tests or one-way analysis of variance (ANOVA) to compare differences in the main study variables among patients with different characteristics, in order to explore the influence of demographic factors. Subsequently, to test the main research hypotheses, we used multiple linear regression analysis to examine the effects of family cohesion and perceived social support on disease identity. Prior to conducting the regression analysis, we assessed the model assumptions, including but not limited to normality, homoscedasticity, linearity, independence of errors, and multicollinearity. The Durbin–Watson test was also used to specifically evaluate the independence of the errors. To test the mediating role of perceived social support, we analyzed the data using Model 4 of the PROCESS macro. The significance of the mediating effect was examined using the Bootstrap method, specifically the bias-corrected percentile approach with 5000 Bootstrap samples. A statistically significant mediating effect (*p* < 0.05) was indicated if the 95% bias-corrected confidence interval did not include zero.

To control for potential confounding factors, clinically relevant variables, including glucose control method, parental educational level (both paternal and maternal), monthly household income, and duration of disease, were included as covariates in the mediation analysis. All categorical covariates were dummy-coded using the following reference groups: “insulin pen” for the binary glucose control method; “junior high school” for parental education (a four-category variable: elementary, junior high school, high school, college and above); “3000–5000” for monthly household income (four categories: <3000, 3000–5000, 5001–10,000, >10,000); and “<5 years” for duration of disease (three categories: <5 years, 5–10 years, >10 years). All resulting dummy variables were simultaneously entered into Model 4.

### 2.6. Ethical Dimension

The study was approved by the Ethics Committee of Zhengzhou University (No. ZZUIRB2025-56). All participants were informed of the study’s purpose and content. Personal information obtained in this study was anonymized and kept strictly confidential. Written informed consent was obtained from patients for their participation. Participants were made aware that they could withdraw from the study at any time.

## 3. Results

### 3.1. Demographic Characteristics of AYA Patients with T1DM

Participants ranged in age from 10 to 24 years, comprising 85 males and 137 females. Additional demographic characteristics are presented in Table 1.

### 3.2. Disease Identity Questionnaire, Perceived Social Support Scale, and Family Cohesion Scale Scores in AYA Patients with T1DM

The mean disease identity score for AYA patients with T1DM was 64.81 ± 14.11. Among them, thirty-five patients (15.8%) scored ≥78 points, indicating high disease identity. Perceived social support scores averaged (57.93 ± 12.88), with 96 patients (43.2%) scoring ≥ 61 points, indicating high perceived social support. Family cohesion scores averaged (47.37 ± 8.91), as shown in Table 2.

### 3.3. Univariate Analysis of Disease Identity in AYA Patients with T1DM

Statistically significant differences in disease identity scores were observed among AYA patients with T1DM across different glucose control methods, parental education levels, monthly household income, and duration of disease (*p* < 0.05), as shown in Table 1.

### 3.4. Findings Regarding the Effects of Family Cohesion and Perceived Social Support on Disease Identity

Table 3 presents the effects of family cohesion and perceived social support on disease identity. In Model 1, the total effect of family cohesion on disease identity was analyzed. The results indicated that family cohesion showed a significant positive association with disease identity (β = 0.615, *p* < 0.001). This model explained 37.9% of the total variance in disease identity (F = 134.052; *p* < 0.001). These findings confirm that family cohesion is positively associated with disease identity among AYA patients with T1DM, thereby supporting Hypothesis 1.

In Model 2, the effect of perceived social support on disease identity was analyzed. The results showed that perceived social support was significantly and positively associated with disease identity (β = 0.728, *p* < 0.001). This model explained 53.0% of the total variance in disease identity (F = 248.164; *p* < 0.001), indicating that perceived social support is positively associated with disease identity among AYA patients with T1DM, which supports Hypothesis 2.

In Model 3, the effect of family cohesion on perceived social support was tested. The analysis revealed that family cohesion was significantly and positively associated with the level of perceived social support (β = 0.679, *p* < 0.001). This model explained 46.1% of the total variance in perceived social support (F = 188.009; *p* < 0.001). The results support the notion that family cohesion is positively associated with perceived social support among AYA patients with T1DM, confirming Hypothesis 3.

To explore the mediating role, family cohesion and perceived social support were simultaneously included in the regression equation in Model 4 to analyze their associations with disease identity. The results showed that after accounting for perceived social support, the strength of the association between family cohesion and disease identity weakened, from β = 0.615 to β = 0.225. This pattern suggests that perceived social support may partly account for the association between family cohesion and disease identity.

**Figure 2:**
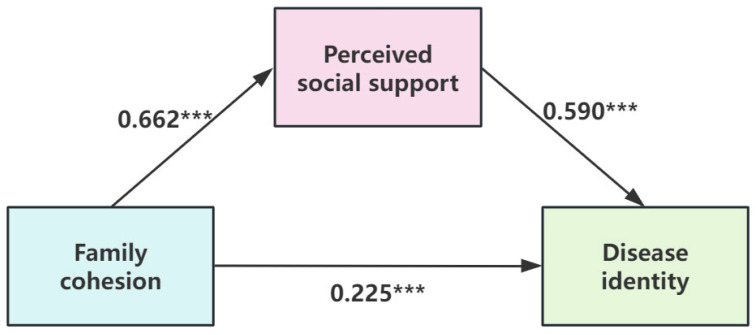
Schematic model illustrating the mediating role of perceived social support between family cohesion and disease identity. Note: All path coefficients shown in the figure are standardized coefficients (β values). *** *p* < 0.001.

To test the hypothesized mediation, a bootstrap analysis was performed using the PROCESS macro (Model 4). The primary findings are reported based on the unadjusted model, with the adjusted model presented as a supplementary analysis for robustness. The results, detailed in Table 4, indicated that perceived social support played a partial mediating role in the association between family cohesion and disease identity. In the primary unadjusted model, the standardized indirect effect was significant (β = 0.391, *p* < 0.001), accounting for 63.6% of the total effect. As illustrated in Figure 2, higher family cohesion was significantly associated with higher perceived social support (β = 0.662, *p* < 0.001), which in turn was associated with stronger disease identity (β = 0.590, *p* < 0.001), while the direct association remained significant (β = 0.225, *p* < 0.001). This pattern of partial mediation held robust in a supplementary analysis that adjusted for key covariates. Therefore, Hypothesis 4 was supported, confirming that the association between family cohesion and disease identity is partially mediated by perceived social support.

## 4. Discussion

This study aimed to examine the role of perceived social support in the association between family cohesion and disease identity among AYA patients with T1DM. The results indicated that family cohesion was not only directly associated with disease identity but also showed an indirect association through perceived social support. The total mediation effect accounted for 63.6%, indicating that perceived social support is a significant factor in the association between family cohesion and disease identity.

### 4.1. Effects of Family Cohesion on Perceived Social Support

The results of this study demonstrate a positive association between family cohesion and perceived social support among AYA patients with T1DM. Specifically, higher levels of family cohesion within the patient’s environment are associated with a greater capacity for perceiving social support. In families with high cohesion, patients perceive stronger parental support, which may enhance their trust in and willingness to rely on the social support system [37]. As a core dimension of the family environment, family cohesion significantly influences patients’ level of perceived social support. Close family bonds provide a foundation for consistent and reliable supportive behaviors, facilitating the smoother internalization of such external support into a stable personal resource for adolescents [38], thereby improving their ability to perceive social support. This finding indicates a direct association between family functioning and patients’ ability to perceive social support. It also suggests that future research explore strengthening family functioning as a potential avenue to enhance patients’ subjective experience of support and, consequently, their capacity to perceive it.

### 4.2. Effects of Family Cohesion on Disease Identity

The findings of this study indicate that family cohesion is positively correlated with disease identity, consistent with the results reported by Merkas et al. [39]. High family cohesion signifies strong emotional bonds, frequent interactions, and a nurturing environment among family members [40], which exerts multiple influences on patients’ disease identity. First, parental emotional support enhances adolescents’ psychological resilience [38], helping patients maintain a balanced perspective on their disease [41]; Second, families actively engage in disease management, helping patients develop healthy habits while integrating disease into daily life. Additionally, these households maintain open communication channels where patients freely express concerns and confusion about their condition. Parents attentively listen and offer guidance, aiding patients in understanding their disease, alleviating fears, and fostering positive disease identity [16].

It is noteworthy that within the collectivist cultural context of China, family dynamics are characterized by patterns of high emotional connectedness and a strong sense of shared responsibility. Within China’s collectivist cultural context, families typically exhibit interaction patterns characterized by high emotional bonding and a strong sense of shared responsibility. In this setting, parents often assume considerable caregiving responsibility for their AYAs’ health [42], with frequent and in-depth emotional communication between parents and AYAs. Consequently, AYAs are more likely to rely psychologically on their parents for support and guidance. This close parent–child interaction pattern establishes the family as the central arena for AYA patients with T1DM to access health-related support, amplifying the impact of family cohesion on patients’ disease adaptation [43]. This stands in marked contrast to the relatively independent parent–child interaction patterns often observed in Western individualistic cultural contexts.

Therefore, this suggests that in the process of improving patients’ disease identity, healthcare professionals could consider the family as a key unit in supportive approaches, rather than solely on the individual patient. For example, the development of family-involved diabetes management programs could be explored, and regular family meetings can be held to discuss challenges related to disease management.

### 4.3. The Mediating Role of Perceived Social Support in the Relationship Between Family Cohesion and Disease Identity

The results of this study indicated that family cohesion among AYA patients with T1DM was indirectly associated with disease identity through perceived social support, with the indirect association accounting for 63.6% of the total association. This finding is highly consistent with the core proposition of social support theory [26], which posits that the supportive resources individuals perceive from social relationships serve as critical buffering factors against the stress associated with chronic disease populations. Specifically, during the campus-life stage, the family functions as the primary emotional support system for AYAs. Enhanced family cohesion helps shape a positive cognitive schema of “being cared for and accepted” in patients [17]. Such a cognitive framework strengthens patients’ subjective perception and utilization capacity of social support resources, thereby elevating their level of perceived social support [44].

High levels of perceived social support prompt patients to re-examine their relationship with the disease, gradually integrate the disease into their self-concept psychologically and embrace their disease identity with an open mindset [25]. They treat the condition as an integral part of life while striving to pursue a meaningful existence alongside it. At this stage, patients develop a clearer perception of their social roles, free from self-doubt and avoidance behaviors triggered by the disease. When receiving assistance from significant others such as family members, friends and teachers, patients are more likely to adopt a positive attitude toward accepting this support and gain greater courage to actively seek social resources [45,46]. This proactive engagement in seeking social support further reinforces the development of an adaptive disease identity, thus forming a virtuous cycle that fosters the establishment of a positive disease identity in patients.

In light of these associations, the findings could inform potential support strategies. For instance, fostering collaboration between family and school might be beneficial in addressing AYAs’ emotional needs, potentially supporting prosocial behavior and the perception of social support. Additionally, for healthcare professionals, these results highlight family cohesion and perceived social support as relevant factors that could be integrated into support frameworks, with the aim of supporting disease management motivation and positive disease identity.

## 5. Limitations and Future Directions

This study has several limitations: (1) The study sample was derived solely from patients at 4 Grade A tertiary hospitals in Zhengzhou City, Henan Province. Variables involved in the study, such as family cohesion, perceived social support and disease identity, are highly influenced by cultural contexts. Chinese family relations and collectivist orientation may differ from the individualist culture in Western countries, and such cultural particularities limit the cross-cultural generalizability of the study results. (2) The cross-sectional design was adopted in this study, making it impossible to infer the causal relationships among variables. Future research needs to further verify the relevant pathways through longitudinal studies. In addition, disease identity itself may be characterized by dynamic development, but this study failed to depict its changing trajectory over time. Long-term follow-up studies in subsequent research will help reveal the evolutionary rules and characteristics of disease identity at different stages of the disease course, thereby providing evidence for developing stage-adapted intervention strategies. (3) The durations of disease data in this study were broad categorical variables rather than continuous variables. This limitation may weaken the ability to detect potential non-linear relationships between duration of disease and disease identity. In our future research, we will address this inadequacy by using precise years of disease duration to further explore the impact of the time since diagnosis on disease identity among patients. (4) This study adopted self-report scales for all measurements, and its results may be affected by the subjective judgment bias of participants. (5) The AYA patients with T1DM Disease Identity Questionnaire used in this study was compiled independently by the research team and has not been systematically validated among populations from different cultural backgrounds. Therefore, there may be cross-cultural biases in terms of its content validity, construct validity and measurement invariance, which to a certain extent limit the external validity of the study conclusions. Future research can further conduct reliability and validity tests and cross-cultural adaptations of this scale among different cultural groups to enhance its applicability. (6) This study focused primarily on psychosocial variables. Given that clinical indicators such as glycated hemoglobin (HbA1c) and diabetic complications were not incorporated, the association between patients’ psychological characteristics and clinical outcomes could not be verified. Future studies could further integrate psychological characteristics and clinical indicators to conduct a more comprehensive investigation into the relationship between these two dimensions.

## 6. Conclusions

The results indicate that for AYA patients with T1DM, a higher level of family cohesion is primarily associated with greater acceptance of disease identity, which may be explained by the creation of an emotionally secure environment that enhances patients’ perception of being understood and accepted by others. From a social cognitive perspective, this finding contributes to our understanding of the mechanisms through which the family system is linked to identity development in patients with chronic diseases. The results suggest a potential pathway linking environmental factors to psychological and behavioral outcomes, offering a theoretical perspective for comprehensive interventions that consider the “family–cognition–identity” chain. In clinical practice, it may be beneficial for healthcare professionals to consider incorporating the assessment and support of family interaction quality into routine care and health education, while also focusing on enhancing patients’ level of perceived social support, as these factors may facilitate the formation of a positive disease identity.

## Figures and Tables

**Table 1:** Univariate Analysis of Disease Identity in AYA Patients with T1DM (n = 222).

Variable		Number of Cases n (%)	Score (Mean ± SD)	t/F Value	*p*-Value	Effect Size (95% CI)
Gender	Male	85 (38.3%)	66.62 ± 14.41	1.508 ^a^	0.133	d = 0.208 [−0.063, 0.479]
	Female	137 (61.7%)	63.69 ± 13.86			
Only child	Yes	26 (11.7%)	67.85 ± 14.27	1.167 ^a^	0.245	d = 0.244 [−0.166, 0.653]
	No	196 (88.3%)	64.41 ± 14.07			
Glucose control method	Insulin pen	142 (64.0%)	63.07 ± 13.04	−2.484 ^a^	0.014	d = −0.347 [–0.623, −0.071]
	Insulin pump	80 (36.0%)	67.91 ± 15.44			
Age	10–14	119 (53.6%)	64.55 ± 12.62	0.047 ^b^	0.954	η^2^ < 0.001 [0.000, 0.006]
	15–19	75 (33.8%)	65.04 ± 17.91			
	20–24	28 (12.6%)	65.32 ± 7.27			
Father’s educational level	Elementary	28 (12.6%)	55.14 ± 12.50	14.481 ^b^	<0.001	η^2^ = 0.166 [0.078, 0.246]
	Junior high school	132 (59.5%)	63.02 ± 13.13			
	High school	39 (17.6%)	72.08 ± 12.51			
	College and above	23 (10.4%)	74.61 ± 13.34			
Mother’s educational level	Elementary	43 (19.4%)	50.30 ± 12.39	45.816 ^b^	<0.001	η^2^ = 0.387 [0.283, 0.465]
	Junior high school	118 (53.2%)	64.40 ± 10.65			
	High school	44 (19.8%)	74.07 ± 10.54			
	College and above	17 (7.7%)	80.47 ± 12.45			
Family residence	Rural	90 (40.5%)	62.87 ± 13.77	2.253 ^b^	0.107	η^2^ = 0.020 [0.000, 0.065]
	Town	77 (34.7%)	64.84 ± 14.15			
	City	55 (24.8%)	67.96 ± 14.29			
Monthly household income (RMB)	<3000	29 (13.1%)	53.76 ± 13.25	24.525 ^b^	<0.001	η^2^ = 0.252 [0.152, 0.335]
	3000–5000	104 (46.8%)	62.76 ± 12.52			
	5001–10,000	66 (29.7%)	66.91 ± 12.79			
	>10,000	23 (10.4%)	82.04 ± 7.39			
Duration of disease	<5	170 (76.6%)	63.95 ± 13.73	8.907 ^b^	<0.001	η^2^ = 0.075 [0.018, 0.144]
	5–10	44 (19.8%)	64.52 ± 13.76			
	>10	8 (3.6%)	84.75 ± 9.91			

Note: ^a^ denotes t-value, ^b^ denotes F-value.

**Table 2:** Scores on the Disease Identity Questionnaire, Perceived Social Support Scale, and Family Cohesion Scale (n = 222, mean ± SD).

Dimension and Total Score	Item	Score (Mean ± SD)
Disease Identity	Total Score	64.81 ± 14.11
	Disease Identity Dimensions	18.77 ± 5.32
	Identity Dimension in Interaction	13.34 ± 3.75
	Identity Choice Dimension	16.94 ± 4.06
	Identity Expression Dimension	15.77 ± 3.99
Perceived Social Support	Total Score	57.93 ± 12.88
	Family Support	11.99 ± 3.55
	Friend Support	12.17 ± 3.55
	Other Support	11.23 ± 2.15
Family cohesion	Total Score	47.37 ± 8.91

**Table 3:** The Effect of Family Cohesion and Perceived Social Support on Disease Identity.

Outcome Variable	Predictors	B	SE	β	*p*	95% CI	
						Lower	Upper
Model 1 Disease Identity	Constant Family cohesion R = 0.615, R^2^ = 0.379, F = 134.052, *p* < 0.001 Durbin-Watson = 2.000	18.653 0.974	4.057 0.084	— 0.615	<0.001 <0.001	10.658 0.809	26.648 1.140
Model 2 Disease Identity	Constant perceived Social Support R = 0.728, R^2^ = 0.530, F = 248.164, *p* < 0.001 Durbin-Watson = 1.856	18.599 0.798	3.005 0.051	— 0.728	<0.001 <0.001	12.676 0.698	24.521 0.898
Model 3 perceived Social Support	Constant Family cohesion R = 0.679, R^2^ = 0.461, F = 188.009, *p* < 0.001 Durbin-Watson = 1.992	11.456 0.981	3.449 0.072	— 0.679	<0.001 <0.001	4.659 0.840	18.253 1.122
Model 4 Disease Identity	Constant Family cohesion perceived Social Support R = 0.747, R^2^ = 0.557, F = 137.833, *p* < 0.001 Durbin-Watson = 1.869	11.427 0.356 0.631	3.517 0.097 0.067	— 0.225 0.576	<0.001 <0.001 <0.001	4.496 0.165 0.498	18.359 0.547 0.763

**Table 4:** Mediation Analysis Results: Primary (Unadjusted) and Robustness Check (Adjusted) Models.

Effect and Path	Primary: Unadjusted Model (Standardized β)	Robustness Check: Adjusted Model (Standardized β)
Total Effect	0.615 ***	0.423 ***
Direct Effect	0.225 ***	0.221 ***
Indirect Effect	0.391 ***	0.202 ***
Proportion Mediated	63.6%	47.8%

Note. Standardized coefficients (β) are reported. The primary analysis is based on the unadjusted model. The adjusted model, controlling for glucose control method, parental educational level, monthly household income, and duration of disease, is presented as a robustness check. Bootstrapped 95% confidence intervals (5000 samples) for the unadjusted/adjusted indirect effects were [0.470, 0.800]/[0.113, 0.312] (in B units), respectively. Proportion mediated = (Indirect Effect/Total Effect) × 100%. *** *p* < 0.001.

## Data Availability

The data set supporting this conclusion is contained in the article and its addenda.

## Public Involvement Statement

No public involvement in any aspect of this research.

## Guidelines and Standards Statement

This manuscript was drafted in accordance with the Strengthening the Reporting of Observational Studies in Epidemiology (STROBE) guidelines, which are applicable to observational research. A complete list of reporting guidelines can be accessed via Epidemiology at http://www.epidem.com/.
